# Supplementary material for: TBX1 and Basal Cell Carcinoma: Expression and Interactions with Gli2 and Dvl2 Signaling
Source: Int J Mol Sci. 2020 Jan 17;21(2):607. doi: 10.3390/ijms21020607 (PMC7014135; doi:10.3390/ijms21020607)
Supplement: Supplementary file 1 [file ijms-21-00607-s001.zip › Legend for supplementary figures.docx]

Legend for supplementary figures.

**Figure S1**

Hematoxylin - Eosin staining of sections from skin biopsies of patients. Panels A-D: these sections are adjacent to those shown in Fig. 2, panels A-C', respectively. Panels E-H: adjacent sections to those shown in Fig. 3, panels A-C'. Scale bars are 300µm.

**Figure S2**

Graphical summaries of quantitative real time PCR of transcripts of the genes indicated. All graphs refer to experiments of *Gli2* knock down in G2N2C cells. Each dot represents a biological replicate. P values are <0.05 for all three genes indicated. Individual P values are indicated. NT-Gli2: non-targeted control siRNA. siGli2: *Gli2*-targeted siRNA.

**Figure S3**

Gene expression analysis in *Tbx1* gene knock down experiments using G2N2C cells. The panel on the left shows examples of reverse transcription PCR of the genes indicated. The graphs on the right are summaries of quantitative real time PCR in repeated experiments (each dot is a biological replicate). *Tbx1* knock down does not change the expression of these genes in a significant manner. Quantitative evaluation of *Dvl2* expression, which is significantly down regulated, is shown in Fig. 4E. NT: non-targeted control siRNA. siTbx1 or Tbx1 KD: siRNA targeting the *Tbx1* transcript. -Ctr: blank.
