# Supplementary figures and images for: TBX1 and Basal Cell Carcinoma: Expression and Interactions with Gli2 and Dvl2 Signaling
Source: Int J Mol Sci. 2020 Jan 17;21(2):607. doi: 10.3390/ijms21020607 (PMC7014135; doi:10.3390/ijms21020607)

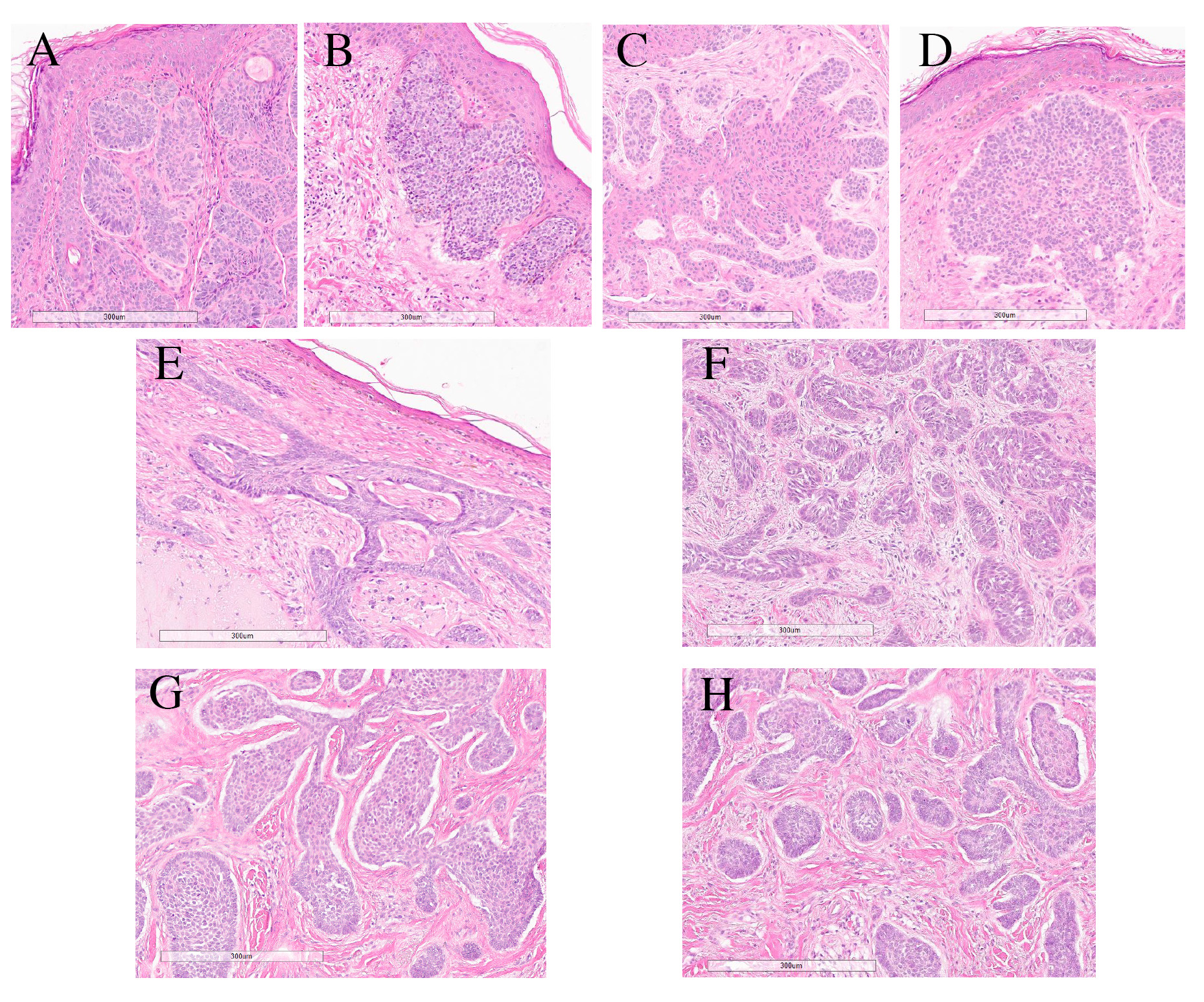

Supplement: Supplementary file 1 [file ijms-21-00607-s001.zip › FigS1H&E.jpg]

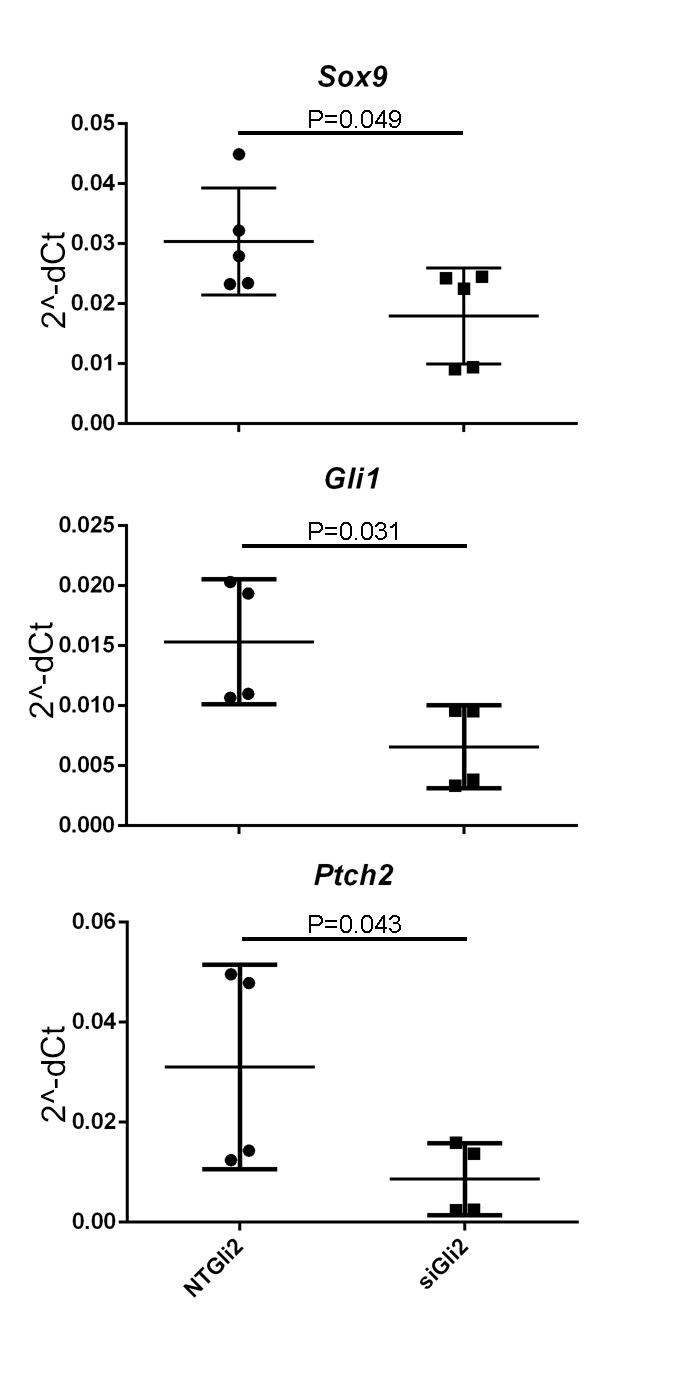

Supplement: Supplementary file 1 [file ijms-21-00607-s001.zip › FigS2 real time.jpg]

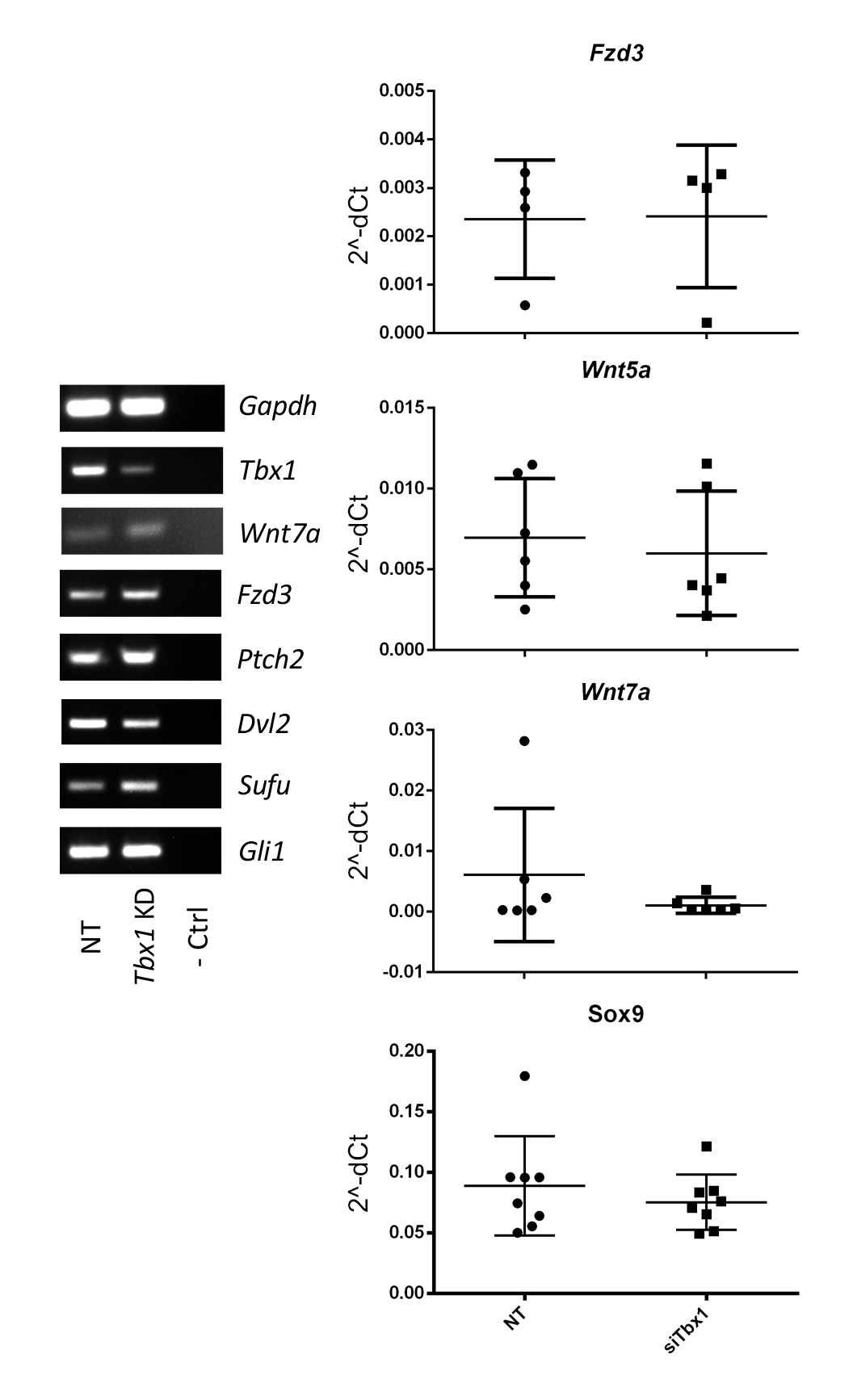

Supplement: Supplementary file 1 [file ijms-21-00607-s001.zip › FigS3 real time.jpg]
